# Protective Regulatory T Cell Immune Response Induced by Intranasal Immunization With the Live-Attenuated Pneumococcal Vaccine SPY1 *via* the Transforming Growth Factor-β1-Smad2/3 Pathway

**DOI:** 10.3389/fimmu.2018.01754

**Published:** 2018-08-02

**Authors:** Hongyi Liao, Xiaoqiong Peng, Lingling Gan, Jiafu Feng, Yue Gao, Shenghui Yang, Xuexue Hu, Liping Zhang, Yibing Yin, Hong Wang, Xiuyu Xu

**Affiliations:** ^1^Key Laboratory of Diagnostic Medicine Designated by the Ministry of Education, Chongqing Medical University, Chongqing, China; ^2^School of Laboratory Medicine, Chongqing Medical University, Chongqing, China; ^3^Department of Ultrasound, The First Affiliated Hospital of Chongqing Medical University, Chongqing, China; ^4^Department of Clinical Laboratory, Mianyang Central Hospital, Mianyang, Sichuan, China; ^5^Department of Laboratory Medicine, The First Affiliated Hospital of Chongqing Medical University, Chongqing, China

**Keywords:** *Streptococcus pneumoniae*, vaccine, protective mechanism, transforming growth factor β1, regulatory T cells

## Abstract

Vaccine effectiveness is mainly determined by the mechanism mediating protection, emphasizing the importance of unraveling the protective mechanism for novel pneumococcal vaccine development. We previously demonstrated that the regulatory T cell (Treg) immune response has a protective effect against pneumococcal infection elicited by the live-attenuated pneumococcal vaccine SPY1. However, the mechanism underlying this protective effect remains unclear. In this study, a short synthetic peptide (P17) was used to downregulate Tregs during immunization and subsequent challenges in a mouse model. In immunized mice, increase in immune cytokines (IL-12p70, IL-4, IL-5, and IL-17A) induced by SPY1 were further upregulated by P17 treatment, whereas the decrease in the infection-associated inflammatory cytokine TNF-α by SPY1 was reversed. P17 also inhibited the increase in the immunosuppressive cytokine IL-10 and inflammatory mediator IL-6 in immunized mice. More severe pulmonary injuries and more dramatic inflammatory responses with worse survival in P17-treated immunized mice indicated the indispensable role of the Treg immune response in protection against pneumococcal infection by maintaining a balance among acquired immune responses stimulated by SPY1. Further studies revealed that the significant elevation of active transforming growth factor β (TGF-β)1 by SPY1 vaccination activated FOXP3, leading to increased frequencies of CD4^+^CD25^+^Foxp3^+^ T cells. Moreover, SPY1 vaccination elevated the levels of Smad2/3 and phosphor-Smad2/3 and downregulated the negative regulatory factor Smad7 in a time-dependent manner during pneumococcal infection, and these changes were reversed by P17 treatment. These results illustrate that SPY1-stimulated TGF-β1 induced the generation of SPY1-specific Tregs *via* the Smad2/3 signaling pathway. In addition, SPY1-specific Tregs may participate in protection *via* the enhanced expression of PD-1 and CTLA-4. The data presented here extend our understanding of how the SPY1-induced acquired Treg immune response contributes to protection elicited by live-attenuated vaccines and may be helpful for the evaluation of live vaccines and other mucosal vaccine candidates.

## Introduction

*Streptococcus pneumoniae*, an important opportunistic pathogen that colonizes the human oral and nasopharyngeal cavities, is the leading cause of pneumonia in the elderly, immunocompromised, and children younger than 5 years (1, 2). Vaccination is an effective means of preventing pneumococcal disease (3). Currently, both injection and the intranasal administration of pneumococcal vaccines are recommended by the WHO (4). However, intranasal administration can stimulate mucosal immune responses, unlike the conventional systemic delivery of vaccines using a needle and syringe. Furthermore, the mucosal delivery of vaccines can induce systemic immunity, similar to that induced by injection-based vaccination. Mucosal vaccination is pain-free, reduces the risk of needle reuse, and reduces the burden on healthcare professionals, among other benefits (5). However, commercial pneumococcal polysaccharide vaccines and conjugate vaccines are constrained by limited serotype coverage, high-cost, and vaccine serotype replacement (6, 7); accordingly, it is necessary to develop novel vaccine candidates who can overcome these disadvantages. Due to the strong antigenicity and comprehensive serotype coverage promised by a wide range of antigenic molecules, whole-cell pneumococcal vaccines are regarded as ideal vaccine candidates (8, 9). Importantly, vaccine effectiveness is mainly dependent on the underlying protective mechanism; therefore, unraveling this mechanism is critical for novel pneumococcal vaccine development.

*Streptococcus pneumoniae* strain SPY1 is a live-attenuated pneumococcal vaccine. We have systematically described the extremely reduced virulence, reliable genetic stability, high safety, and excellent protection against pneumococcal infection in a mouse model (10). Humoral and Th2–Th17 T cell immune responses are indispensable for the protection induced by SPY1 (11). We have also surprisingly detected a protective role of the regulatory T cell (Treg) immune response elicited by SPY1, which has not previously been described for *S. pneumoniae* vaccines (11). These findings highlight the importance of the development of novel pneumococcal vaccines that can induce a protective Treg response, which is vital for the maintenance of immune homeostasis as well as for limiting infection-associated inflammation and facilitating the resolution of tissue damage post-infection. However, the mechanism underlying the activation of the SPY1-induced Treg response is still unknown.

As a pleiotropic cytokine, transforming growth factor β (TGF-β) is essential for the differentiation of Tregs in innate immunity (12). TGF-β could induce the expression of Foxp3 and the conversion of CD4^+^CD25^−^ T cells to CD4^+^CD25^+^ T cells, promoting Treg proliferation and subsequent immunosuppression (13–15). In our previous study, a short peptide P17 (KRIWFIPRSSWYERA) was introduced to inhibit the production of TGF-β1 and to downregulate Tregs (11, 16, 17). As expected, treatment with P17 significantly impairs the effectiveness of SPY1 in colonization and invasive infection models, suggesting the importance of SPY1-induced TGF-β1 for the protective Treg immune response in acquired immunity. Several signaling pathways, including Smad-dependent and Smad-independent pathways, have potential roles in the activation of Tregs mediated by activated TGF-β1, and the specific functional pathway varies among experimental models (18–21). Previous research has shown that the percentage of Tregs is elevated in SPY1-vaccinated mice; however, signaling mechanism by which TGF-β1 mediates the differentiation of Tregs is unknown.

In this study, the mechanism underlying the protective Treg response activated by vaccination with SPY1 was explored. The inhibition of TGF-β1 dramatically attenuated the SPY1-induced protection against pulmonary injuries caused by pneumococcal colonization. In addition, SPY1-induced TGF-β1 is essential for the balance among systemic protective immune responses triggered by SPY1 vaccination. The activation of the TGF-β1-Smad2/3 signaling pathway is responsible for the generation of Tregs, which are involved in SPY1 protection *via* the elevated expression of CTLA-4 and PD-1. These findings and those of our previous studies provide insight into the proximal mechanism mediating the protection elicited by the SPY1-induced acquired Treg immune response and may contribute to a more comprehensive evaluation of live vaccines and other mucosal vaccines.

## Materials and Methods

### Mice

Female C57BL/6 mice (6–8 weeks) were purchased from the animal center of Chongqing Medical University, Chongqing, China. Mice were kept under specific pathogen-free conditions at the animal facilities of Chongqing Medical University during the time of the experiments. All animal experiments were performed in accordance with the guidelines of the Institutional Animal Care and Use Committee of Chongqing Medical University.

### Bacterial Strains and Immunogen Preparation

*Streptococcus pneumoniae* strain NCTC 7466 (D39, serotype 2) was obtained from the National Collection of Type Cultures (NCTC; London, UK). The *S. pneumoniae* clinical isolate CMCC 31693 (serotype 19F) was obtained from the National Center for Medical Culture Collections (CMCC; Beijing, China). SPY1 is a novel live-attenuated *S. pneumoniae* vaccine candidate strain with definite serotype-independent protection against pneumococcal infection (10), and its basic protective mechanism has been described in our previous research (11). All pneumococcal strains were grown in casein-based medium with yeast extract (C + Y medium) or on Columbia sheep blood agar plates at 37°C in 5% CO_2_. To prepare the immunogens, SPY1 was grown at 37°C in 5% CO_2_ in C + Y medium to approximately 2 × 10^8^ CFU/ml. After centrifugation and washing (twice), sediments were resuspended in sterile phosphate-buffered saline (PBS). The final vaccine mixture for routine immunization contained 1 × 10^8^ CFU of SPY1 and 1 µg of adjuvant cholera toxin (CT; Sigma-Aldrich, St. Louis, MO, USA) per 20-µl dose.

### Immunization of Mice and Challenge

**Figure 1:**
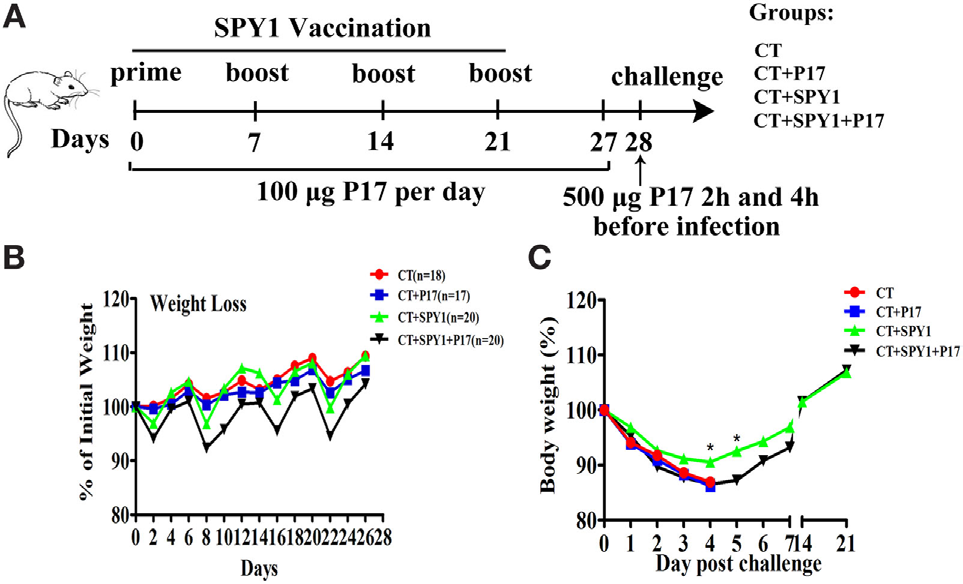
Regulatory T cell (Treg) downregulator P17 eliminates SPY1-elicited protection against invasive *S. pneumonia* infection. **(A)** Mice were intranasally immunized with SPY1 plus CT or with CT alone for four times at 1-week intervals. During the whole vaccination progress of 28 days in all, half of mice from group CT + SPY1 were randomly chosen to be intraperitoneally injected with 100 µg of peptide P17 daily and the other half of mice in group CT + SPY1 received sterile phosphate-buffered saline (PBS) as control. Similarly, mice from CT group were also injected with P17 or PBS daily. On day 28, mice were intranasally challenged with pneumococcal strains. **(B)** Mice body weight were monitored every other day during the vaccination procedure, and the weight data were expressed as the percentages of initial mice weights measured at day 0 of vaccination. On day 28, mice were intranasal challenged with 1 × 10^8^ CFU of pneumococcal strain D39, and the body weights of live mice were monitored for consecutive 21 days after infection **(C)**. *p < 0.05.

C57BL/6 mice were anesthetized with ethyl ether and then intranasally administered the vaccine (group CT + SPY1) or adjuvant alone (group CT) four times at 1-week intervals. One week after the last vaccination, mice were intranasally challenged with either strain 19F (1 × 10^8^ CFU) or strain D39 (1 × 10^8^ CFU). To investigate the roles and related mechanisms of the SPY1-induced Treg immune response, as shown in Figure 1A, during the whole vaccination period of 28 days, half of the mice in the CT + SPY1 group were randomly chosen and treated with an intraperitoneal injection of 100 µg of peptide P17 daily to downregulate Tregs (group CT + SPY1 + P17), as described previously (11). The other half of mice in the CT + SPY1 group received sterile PBS as a control. Similarly, mice from CT group were also injected with P17 or PBS daily. In addition to 28 days of P17 treatment, on the day of challenge, the P17-treated mice received 500 µg of P17 twice at 2 and 4 h before challenge (Figure 1A). The peptide P17 (KRIWFIPRSSWYERA; purity >95%, as determined by high performance liquid chromatography) was synthesized by GL Biochem (Shanghai, China).

### Flow Cytometry

Mouse lungs were removed and cell suspensions were prepared. For Treg (CD4^+^CD25^+^Foxp3^+^) detection, lung cells were first incubated with CD16/CD32 and stained with anti-mouse CD4-FITC (clone RM4-5; eBioscience, San Diego, CA, USA) and anti-mouse CD25-APC (clone PC61.5; eBioscience). Then, these cells were fixed and permeabilized in Fix/Perm buffer and subsequently incubated with anti-mouse Foxp3-PE (clone FJK-16S; eBioscience), according to the instructions provided with the Mouse Regulatory T Cell Staining Kit (eBioscience) for the intracellular Foxp3 analysis. To analyze the expression of PD-1 and CTLA-4, cells were stained with anti-mouse CD279 (PD-1)-APC/Cy7 (clone 29F.1A12; BioLegend, San Diego, CA, USA) and anti-mouse CD152 (CTLA-4)-PerCP/Cy5.5 (clone UC10-4B9; BioLegend). All the samples were analyzed using a Becton Dickinson FACSCalibur flow cytometer (Franklin Lakes, NJ, USA).

### RNA Extraction and Quantitative Real-Time PCR

Mouse lungs were removed at different time points after pneumococcal 19F challenge, and total RNA was extracted using RNAiso Plus reagent (Takara Bio, Dalian, China) following the manufacturer’s instructions. For reverse transcription, 1 µg of total RNA was reverse-transcribed into cDNA, and the PrimeScript™ RT Reagent Kit (Takara Bio) was used for reverse transcription. PCR amplification was performed on the Bio-Rad CFX96 Real-Time system using SYBR Premix Ex Taq™ (Takara Bio). The 2^−ΔΔCt^ method was used to determine the specific Ct value of each target gene. Quantitative real-time PCR for each target gene were repeated three times. The PCR primers were synthesized by Sangon (Shanghai, China) and the sequences are listed in Table S1 in Supplementary Material. All genes were murine in origin.

### Protein Extraction and Western Blot Analysis

Lungs were collected at different time points after pneumococcal 19F challenge, and proteins were extracted by adding the appropriate volume of whole-cell lysates [RIPA buffer (Beyotime, Shanghai, China): PMSF: phosphatase inhibitor (BioTools, Jupiter, FL, USA) = 100:10:1]. For the detection of Smad2/3, phosphor-Smad2/3, and Smad7 at the protein levels, the same concentrations of total cellular extracts were separated by 10% SDS-PAGE, subjected to electrophoresis, and transferred to PVDF membranes (Millipore, Bedford, MA, USA) by electroblotting. After they were blocked with 5% skim milk for 2 h at room temperature, membranes were incubated overnight at 4°C with primary antibodies, including anti-mouse β-actin, anti-mouse Smad2/3, anti-mouse phosphor-Smad2/3 (Cell Signaling Technology, Danvers, MA, USA), and anti-mouse Smad7 antibodies (Abcam, Cambridge, UK), followed by incubation with an HRP-conjugated secondary antibody for 1 h at room temperature. The Enhanced Chemiluminescence (ECL) Western Blotting System (GE Healthcare, Little Chalfont, UK) was used to detect the target bands. The band intensities were quantified using Quantity One (Bio-Rad Laboratories, Hercules, CA, USA).

### Cytokine Assays

Mouse lungs and spleens were fully homogenized and centrifuged, and the supernatants were collected for cytokine measurement. In addition, mouse splenocytes were plated in 24-well tissue culture plates in 1 ml of DMEM with 10% fetal calf serum (HyClone, Logan, UT, USA) (2 × 10^7^ cells/well) after red blood cells were removed by hemolysis. Cultured splenocytes were stimulated with 70% ethanol-inactivated SPY1 (equivalent to 10^7^ CFU/ml), and the supernatants were harvested at different time points for cytokine measurement. Levels of IL-6, TNF-α, IL-12p70, IFN-γ, IL-4, IL-5, IL-17A, and IL-10 in homogenate supernatants and splenocyte supernatants were measured using an enzyme-linked immunosorbent assay (ELISA) kit (BioLegend) in accordance with the manufacturer’s protocols. Samples were diluted when required.

### Immunofluorescence Assay

At 24 h post-pneumococcal 19F challenge, mouse lungs were removed and fixed in 4% paraformaldehyde for 24 h and permeated in 20% sucrose for 24 h. Then, the tissues were frozen in OCT at −20°C and cryo-cut for slides. For Smad2/3 staining, after thawing and blocking with goat serum and subsequent washing with PBS, slides were covered with an anti-Smad2/3 antibody (Cell Signaling Technology) at 4°C overnight, followed by incubation with Fluorescein-Conjugated Goat Anti-Rat IgG (ZSGB-bio, Beijing, China). DAPI was used to stain cell nuclei for 2 h at room temperature in the dark. The cell morphology and fluorescence intensities were observed using an ECLIPSE 80i microscope equipped with a Nikon INTENSILIGHT C-HGFI. The percentages of lung cells with positive Smad2/3 expression were calculated by counting 100 cells per slide (three mice per group).

### Lung Histology and Immunohistochemistry

On day 7 after the last immunization, mice were intranasally challenged with 1 × 10^8^ CFU of pneumococcal strain 19F. Mice were sacrificed and lung tissues were removed at 6, 12, and 24 h post-infection. After fixation in buffered 10% formalin, lungs were sectioned and embedded in paraffin, and 5-µm sections were cut. The sections were stained with hematoxylin and eosin (Sigma-Aldrich) and then examined using a light microscope. The degrees of peribronchial inflammation were graded semi-quantitatively following previously described methods (22).

For immunohistochemistry, sections were retrieved in citrate buffer for 5 min. After natural cooling, sections were incubated with 3% H_2_O_2_ and washed three times with PBS, followed by incubation with an anti-FOXP3 antibody (BioLegend) and Rabbit anti-TGF-β1 polyclonal antibody (OmnimAbs, Alhambra, CA, USA) and treatment with streptavidin horseradish peroxidase chemistry according to standard protocols. The mean IODs (integral optical density) of TGF-β1 expression were measured using Image-Pro Plus (Media Cybernetics, Silver Spring, MD, USA).

### Data Analysis and Statistics

Statistical analyses were performed using GraphPad Prism 5 (GraphPad Software, La Jolla, CA, USA). Unpaired Student’s *t*-tests were used to compare two independent groups. One-way ANOVA was utilized for multiple comparisons. Differences at *p* < 0.05 were considered statistically significant.

## Results

### Treg Downregulator P17 Eliminates SPY1-Elicited Protection Against Invasive *S. pneumoniae* Infection

In this study, female C57BL/6 mice were intranasally immunized according to the vaccination schedule described in Figure 1A. During the vaccination process, half of the immunized mice were intraperitoneally injected with 100 µg of the short synthetic peptide P17 to downregulate Treg activity, and the body weights of all mice were observed every other day. Compared with the CT group, mice in the SPY1 group lost more body weight in the first several days after the first vaccination, and body weight in the of SPY1 group recovered quickly to the level of the CT group (Figure 1B). However, we observed greater body weight loss in mice in the CT + SPY1 + P17 group than in the CT + SPY1 group during the vaccination period (Figure 1B), which may be due to the disruption of the immune regulatory response caused by P17 administration.

On day 28, mice were intranasally challenged with *S. pneumoniae* strain D39. Greater body weight loss was observed in non-immunized mice than in immunized mice on day 4 post-challenge (Figure 1C). Beginning at day 5 post-D39 infection, body weights of mice belonging to the SPY1-immunized group began to increase, finally recovering to the weights recorded before infection, illustrating the protection elicited by SPY1, to some extent (Figure 1C). The body weights of mice in the P17-treated SPY1-immunized group also recovered on day 5 post-infection; however, the degree of body weight increase was significantly lower than that in the SPY1-immunized group (Figure 1C). More importantly, compared with other mouse groups, SPY1-immunized mice survived for longer after lethal *S. pneumoniae* strain D39 infection (Figure S1 in Supplementary Material), consistent with our previous results (11). Collectively, these data demonstrated that the Treg downregulator P17 can markedly eliminate SPY1-elicited protection against invasive *S. pneumoniae* infection, highlighting the importance of the protective Treg immune response.

### P17 Treatment Disturbs the Immune Responses Triggered by SPY1 Vaccination

**Figure 2:**
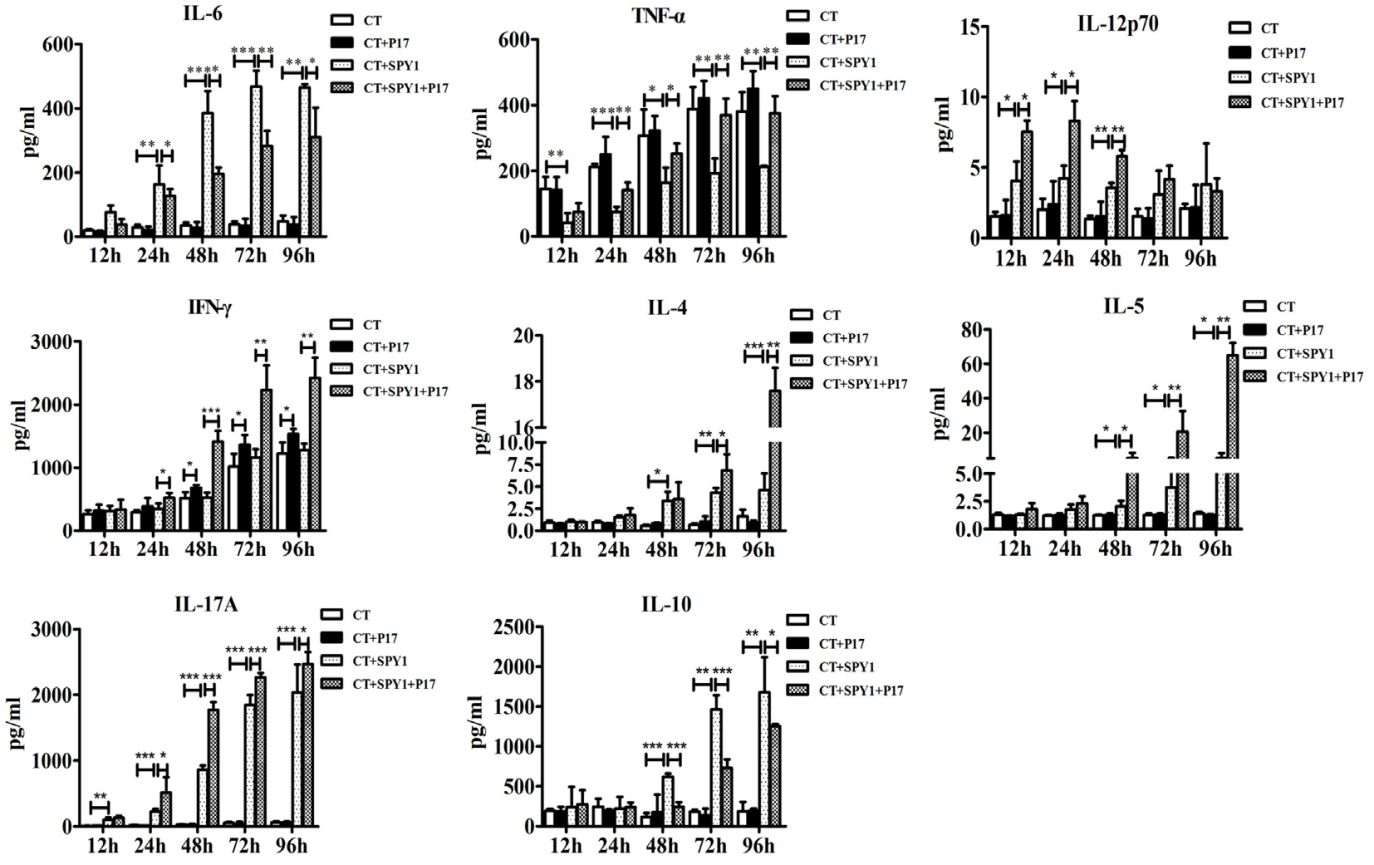
P17 treatment strikes the balance among systemic protective immune responses triggered by SPY1 vaccination. On day 7 after the last immunization, mice splenocytes were isolated and stimulated with 70% ethanol-inactivated SPY1. Splenocytes treated with Concanavalin A served as positive control. Concentrations of cytokines including IL-6, TNF-α, IL-12p70, IFN-γ, IL-4, IL-5, IL-17A, and IL-10 in supernatants of stimulated splenocytes were analyzed by enzyme-linked immunosorbent assay. All data were presented as the mean ± SD of three independent experiments. *p < 0.05; **p < 0.01; ***p < 0.001.

To further investigate the influence of P17 administration on the specific immune responses induced by SPY1, concentrations of immune and inflammatory cytokines in mouse splenocyte supernatants after stimulation by inactivated SPY1 were determined on day 7 after the last immunization. Coincident with our previous results (11), stimulation with inactivated SPY1 induced higher levels of IL-6, IL-12p70, IL-4, IL-5, and IL-17A in immunized mice than in non-immunized mouse (Figure 2). Elevated levels of IL-10 in immunized mice indicated the activation of an immunoregulatory response elicited by SPY1 (Figure 2). We detected a disturbance in the SPY1-activated immune responses in P17-treated immunized mice, which was attributed to the inhibition of immunosuppressive Tregs by P17. The increase in immune cytokines (IL-12p70, IL-4, IL-5, and IL-17A) induced by SPY1 were further upregulated by P17 treatment, whereas the decrease in the infection-associated inflammatory cytokine TNF-α by SPY1 was reversed. Furthermore, P17 also inhibited the increase in the immunosuppressive cytokine IL-10 and inflammatory mediator IL-6 (23) in immunized mice (Figure 2).

### P17 Treatment Significantly Impairs SPY1-Induced Protection Against Pulmonary Injury Caused by Pneumococcal Colonization

**Figure 3:**
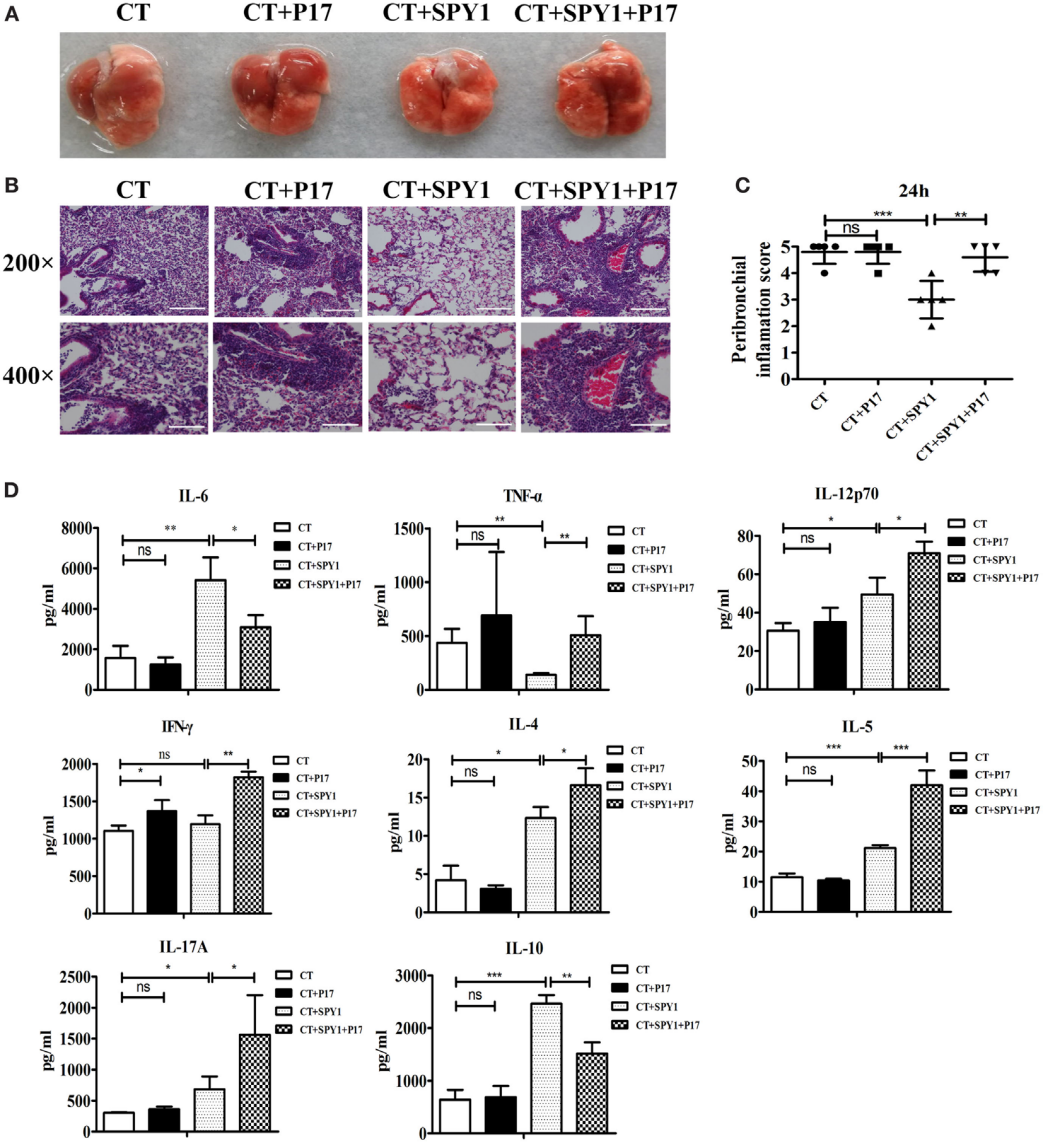
P17 treatment significantly impairs the SPY1-induced acquired protection against pulmonary injury caused by pneumococcal colonization. On day 7 after the last immunization, mice were intranasal challenged with 1 × 10^8^ CFU of pneumococcal strain 19F. Mice were sacrificed and lung tissues were removed at 24 h post-intranasal infection with pneumococcal strain 19F. **(A)** Lungs appearance post-infection. **(B)** Pathological analyses were done by hematoxylin and eosin staining, with lung sections examined under light microscopy at 200× (scale bar = 100 µm) and 400× (scale bar = 50 µm) magnification. **(C)** Scores of peribronchial inflammations were semi-quantitatively graded, and data were shown as mean ± SD of scores of five mice per group. **(D)** Concentrations of cytokines including IL-6, TNF-α, IL-12p70, IFN-γ, IL-4, IL-5, IL-17A, and IL-10 in lung homogenates collected at 48 h post-infection were detected by enzyme-linked immunosorbent assay, and the data were shown as mean ± SD (each experiment was individually performed three times). *p < 0.05; **p < 0.01; ***p < 0.001.

Previously, we established the negative effect of P17 on SPY1-specific protection against *S. pneumoniae* infection, including improved survival rates and reduced bacterial loads in the nasopharynx and lungs (11). In this study, to explore the protective roles of the SPY1-stimulated Treg immune response in more detail, differences in mouse pulmonary damage after pneumococcal colonization among groups were evaluated based on lung morphological observations and histopathological analyses. Moderate inflammatory cell recruitment to the pulmonary interstitium around airways and blood vessels and slight damage to alveolar structural integrity were observed in SPY1-immunized mice post-intranasal challenge with pneumococcal strain 19 F (Figures 3A,B; Figure S2 in Supplementary Material). By contrast, more severe pulmonary injuries were found in the CT control group, with extensive inflammatory cell infiltration in peribronchial as well as perivascular spaces and in the alveoli, disappearance of alveolar structural integrity, and obvious hemorrhage. Similar serious pulmonary injuries were also detected in P17-treated immunized mice, since the immune modulatory response was markedly suppressed. Consistent with these results, compared with other groups, a remarkably lower peribronchial inflammation score was observed in SPY1-immunized mice (Figure 3C). Simultaneously, pulmonary levels of cytokines representing different immune responses triggered by SPY1 vaccination were evaluated. As shown in Figure 3D, compared with non-immunized mice, a decrease in the inflammatory cytokine TNF-α and increase in immune cytokines, including IL-6, IL-12p70, IL-4, IL-5, IL-17A, and IL-10, were found in immunized mouse lung homogenates, indicating elevated, but controlled SPY1-induced immune responses, which are beneficial for fighting against pneumococcal colonization. By contrast, due to the downregulation of the immune-suppressive cytokine IL-10 and consequent immune imbalance caused by P17 treatment, mice showed drastic and uncontrolled pulmonary immune responses as well as infection-associated inflammation, characterized by even higher concentrations of TNF-α, IL-12p70, IFN-γ, IL-4, IL-5, and IL-17A (Figure 3D), consistent with the degrees of pulmonary injury described above.

### SPY1 Vaccination Activates the Expression of the Treg Molecule FOXP3

**Figure 4:**
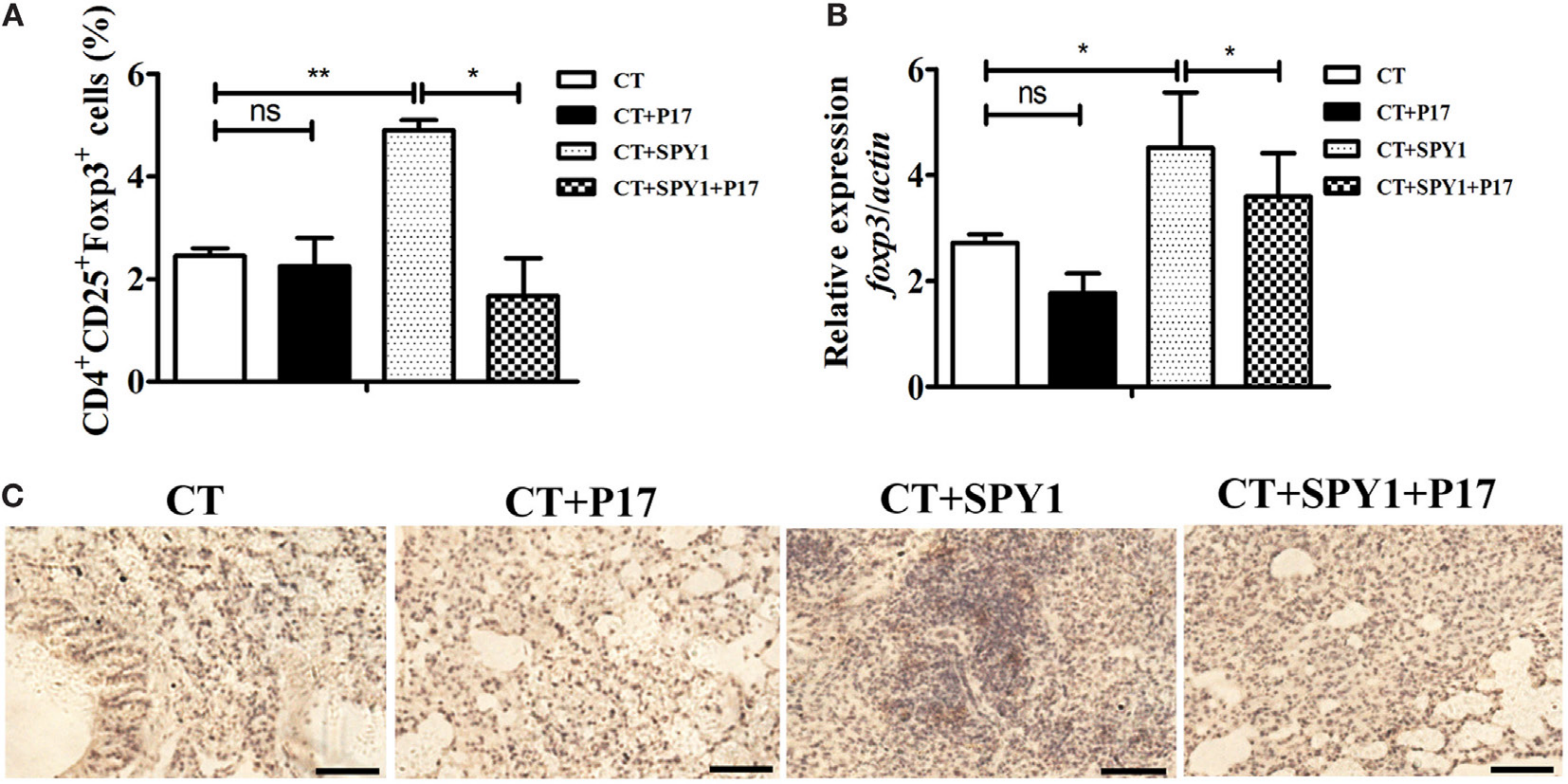
SPY1 vaccination activates the expression of regulatory T cells (Tregs) molecule Foxp3. **(A)** On day 7 after the last immunization, mice lungs were aseptically removed and homogenized, and single lung cell was stained with anti-mouse CD4-FITC and anti-mouse CD25-APC, followed by anti-mouse Foxp3-APC according to the manufacturer’s instructions. Cells were analyzed using a Becton Dickinson FACSCalibur flow cytometer then. The percentages of CD4^+^ T cells which were CD25^+^Foxp3^+^ Treg were calculated. **(B)** On day 7 after the last immunization, mice lungs were aseptically removed, and total RNA were extracted. Expression of *foxp3* mRNA was analyzed by quantitative real-time PCR. Data were shown as mean ± SD from three independent experiments. **(C)** On day 7 after the last immunization, mice lungs were aseptically removed, and lung sections were stained with anti-FOXP3 antibody followed by streptavidin horseradish peroxidase chemistry. Then, the sections were examined under light microscopy at 100× magnification. Scale bar = 100 µm. Images were representative of staining observed in the lungs of mice within the group (*n* = 4–6 mice). *p < 0.05; **p < 0.01.

In our previous study, we were surprised to detect a protective role of the SPY1-specific Treg immune response during pneumococcal infection (11). In this study, to comprehensively analyze the mechanism underlying the activation of Tregs by vaccination, the expression of the transcription factor Foxp3, a characteristic molecule in the Treg immune pathway, was first examined. Flow cytometry results showed a significant increase (*p* < 0.01) in CD4^+^CD25^+^Foxp3^+^ T cells in immunized mouse lungs; however, P17 treatment could reverse the increase in Foxp3-positive cells to a level similar to that in the CT control group (Figure 4A). More importantly, the *foxp3* mRNA level was significantly higher in SPY1-immunized mice than in the CT control group, and the upregulation was reversed by P17 treatment (Figure 4B). The inhibition of FOXP3 in SPY1-vaccinated mice by P17 treatment was also verified by immunohistochemical staining of lung tissues (Figure 4C). Thus, these results demonstrated that SPY1 triggers a FOXP3 response.

### SPY1 Vaccination Stimulates TGF-β1 Production

**Figure 5:**
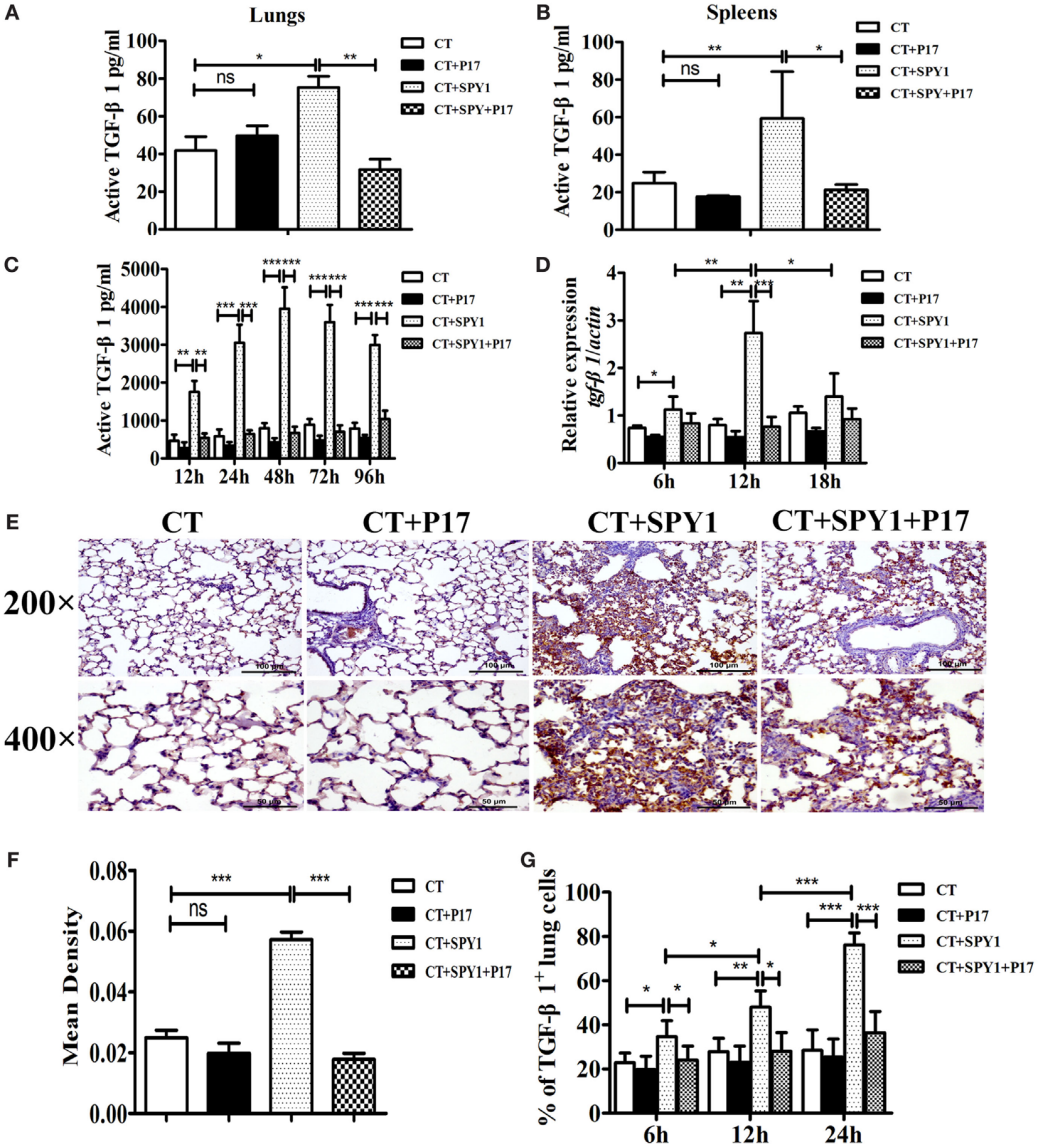
SPY1 vaccination stimulates the production of transforming growth factor β (TGF-β)1. On day 7 after the last immunization, mice were sacrificed and concentrations of active TGF-β1 in lungs **(A)** and spleens **(B)** were determined by enzyme-linked immunosorbent assay (ELISA). **(C)** Splenocytes were isolated and stimulated with 70% ethanol-killed SPY1, and the level of active TGF-β1 in splenocytes supernatants was also detected by ELISA. **(D)** Mice were intranasal challenged with 1 × 10^8^ CFU of pneumococcal strain 19F on day 7 after the last immunization, and expression of *tgf-*β*1* mRNA in lungs were analyzed by quantitative real-time PCR at 6, 12, and 18 h post-infection, respectively. **(E)** At 24 h post-pneumococcal 19F infection, immunohistochemical staining with anti-TGF-β1 polyclonal antibody was utilized to detect expression of TGF-β1 in lung tissues. Lung sections were examined under light microscopy at magnification 200× (scale bar = 100 µm) and 400× (scale bar = 50 µm). **(F)** The mean IODs of TGF-β1 expression were measured and calculated by Image-Pro Plus. Data were shown as mean ± SD of IODs of five mice per group. **(G)** Expressions of TGF-β1 in mice lungs at 6, 12, and 24 h post 19F infection were determined by immunohistochemical staining, and percentages of lung cells with positive TGF-β1 expressions were calculated by counting 100 cells of each lung section with three slides per time point (three mice per group). Data were shown as the mean ± SD from three independent experiments. *p < 0.05; **p < 0.01; ***p < 0.001.

Transforming growth factor β is a vital cytokine for the differentiation of Tregs (14). To identify the mechanism underlying the generation of SPY1-specific Tregs, the production of TGF-β1, one of the three isoforms expressed mainly in the immune system in immunized mice, was evaluated. As shown in Figures 5A,B, substantial increases in the concentrations of active TGF-β1 in lung homogenates as well as spleen homogenates in immunized mice were observed by ELISA. Splenocytes from mice immunized with SPY1 expressed significantly more active TGF-β1 in response to inactivated SPY1 *in vitro* than that of splenocytes from CT-treated control mice (Figure 5C), illustrating the stimulation of TGF-β1. Further verifying the production of SPY1-stimulated TGF-β1, *tgf-β1* mRNA, levels were higher in immunized mouse lungs after intranasal infection with pneumococcal strain 19F than in the CT-treated control group, reaching peak levels at 12 h post-infection (Figure 5D). Immunohistochemical staining of mouse lung tissues with TGF-β1 antibodies also proved the activation of TGF-β1 by vaccination with SPY1 (Figures 5E,F; Figure S3 in Supplementary Material), with a remarkable increase in the percentage of TGF-β1-positive lung cells from SPY1-immunized mice in a time-dependent manner (Figure 5G). More importantly, all of the trends indicating an increase in active TGF-β1 in immunized mice were obviously inhibited by the administration of P17, providing further evidence that SPY1 vaccination could stimulate the production of TGF-β1.

### TGF-β1-Smad2/3 Signaling Participates in the Generation of SPY1-Specific Tregs

**Figure 6:**
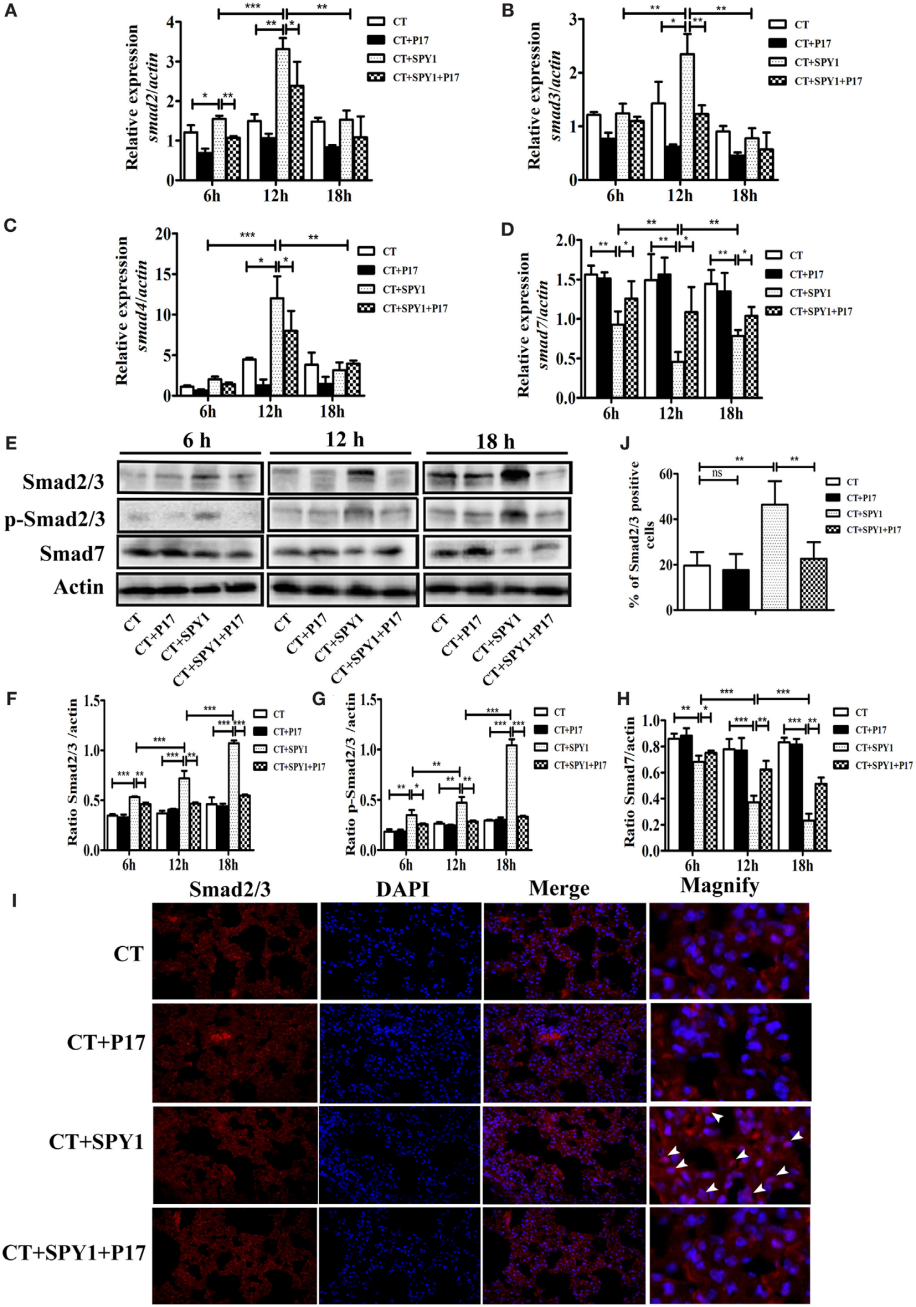
Transforming growth factor β (TGF-β)1-Smad2/3 signaling participates in generation of SPY1-specific regulatory T cells (Tregs). Mice were intranasal challenged with pneumococcal strain 19F on day 7 after the last immunization, and the lungs were aseptically removed at 6, 12, and 18 h post-infection. **(A–D)** Expression of *smad2, smad3, smad4*, and *smad7* in lungs were analyzed by quantitative real-time PCR. The productions of Smad2/3, phosphor-Smad2/3, and Smad7 in lungs were, respectively, determined by western blot **(E)** and the related band intensities were shown in histograms **(F–H)**. **(I)** The expression of Smad2/3 in lungs at 24 h post-infection was examined by immunofluorescence assay with anti-Smad2/3 antibody. Lung sections were examined under light microscopy at magnification 400×. **(J)** Statistical analysis of the percentages of lung cells with positive Smad2/3 expression was performed by counting 100 cells per slide (three mice per group). Data were shown as the mean ± SD from three independent experiments. *p < 0.05; **p < 0.01; ***p < 0.001.

Classic TGF-β-inducing intracellular signaling is mediated by SMAD family proteins, and the differentiation of Tregs from naïve T cells is mainly regulated by the TGF-β/Smad pathway (24). To explore the specific signaling pathway involving in the differentiation of SPY1-induced Tregs, dynamic changes in *Smad* mRNA in mouse lungs post-pneumococcal infection were detected. As shown in Figures 6A–C, compared with CT-treated mice, the expression levels of *smad2, smad3*, and *smad4* mRNA were substantially higher in immunized mice at 6 and 12 h post-pneumococcal strain 19F infection and were also significantly higher than those in P17-treated SPY1-vaccinated mice. A relatively low level of *smad7*, which is a negative regulator in the TGF-β signaling pathway, was detected in immunized mice (Figure 6D). Moreover, P17 treatment significantly upregulated the expression of *smad7* in the immunized group, since P17 injection could block TGF-β signaling. Furthermore, total Smad2/3, phosphorylated Smad2/3, and Smad7 protein expression levels were analyzed by western blotting (Figures 6E–H). Consistent with the observed mRNA expression changes, the expression of Smad2/3 was upregulated in immune mice and downregulated in P17-injected mice at 12 and 18 h post-infection. The stimulation of the TGF-β1/Smad pathway in mouse lungs by SPY1 vaccination was also established by an immunofluorescence assay, indicating significant differences in the percentages of cells expressing Smad2/3 between immunized mice and P17-treated immunized mice (Figures 6I,J). We further evaluated whether other SMAD-independent pathways were involved in the stimulation of SPY1-specific Tregs, but the mRNA expression levels of various signaling molecules including *p38 mapk, akt, mtor*, and *pi3k*, did not differ significantly among groups (data not shown). Taken together, these results confirmed that SPY1 vaccination could activate the TGF-β1-Smad2/3 signal pathway to induce a SPY1-specific Treg response and, as a consequence, protection during pneumococcal infection.

### SPY1 Vaccination Stimulates the Elevated Expression of PD-1 and CTLA-4 on Tregs in Immunized Mice

**Figure 7:**
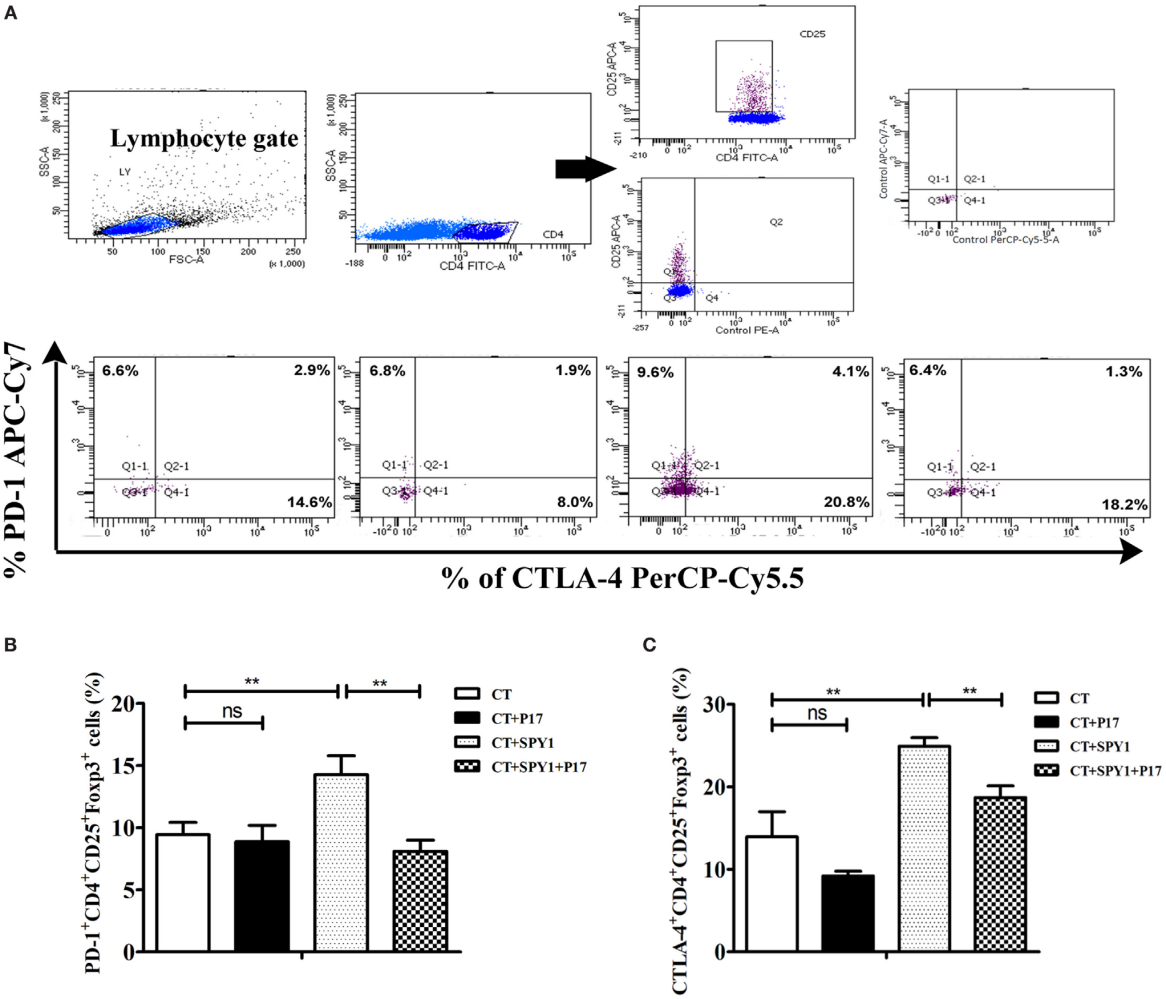
SPY1 vaccination stimulates elevating expression of PD-1 and CTLA-4 on regulatory T cells (Tregs) in immune mice. **(A)** Mice were intranasal challenged with pneumococcal strain 19F on day 7 after the last immunization, and mice lungs were removed on day 3 post-infection. The relative expression of PD-1 and CTLA-4 on CD4^+^CD25^+^Foxp3^+^ cells were analyzed by flow cytometry with antibodies including anti-mouse CD279(PD-1)-APC/Cy7, anti-mouse CD152(CTLA-4)-PerCP/Cy5.5, anti-mouse CD4-FITC, anti-mouse CD25-APC, and anti-mouse Foxp3-PE, respectively. The procedure was as describe in Section “Materials and Methods.” Percentages of PD-1^+^CD4^+^CD25^+^Foxp3^+^ cells **(B)** and CTLA-4^+^CD4^+^CD25^+^Foxp3^+^ cells **(C)** in mice lung of triplicate samples were calculated. Data were shown as the mean ± SD. **p < 0.01.

As an immune response balance factor, Tregs play roles in immunosuppression *via* multiple Treg-associated cell surface molecules and secreted molecules (25). Therefore, to investigate the specific effectors of SPY1-induced Tregs, the expression of PD-1 and CTLA-4 on CD4^+^CD25^+^Foxp3^+^ cells was examined by flow cytometry (Figure 7A). As shown in Figures 7B,C, the percentages of PD-1^+^CD4^+^CD25^+^Foxp3^+^ and CTLA-4^+^CD4^+^CD25^+^Foxp3^+^ cells were both significantly higher in SPY1-immunized mice than in the CT control group, while treatment with P17 (CT + SPY1 + P17 group) reversed the upregulation of immunosuppressive factors. Taken together, these results indicated that PD-1 and CTLA-4 are involved in the SPY1-specific immune modulatory response.

## Discussion

Investigations of the mechanism underlying immune protection are crucial for novel vaccine development. We have previously detected protective roles of the acquired Treg response induced by the novel live-attenuated pneumococcal vaccine SPY1 during pneumococcal infection. In this study, we clarified the mechanism mediating the protective Treg response.

Pneumococcal conjugate vaccines reduce the risk of nasopharyngeal colonization by the serotypes included in the vaccine (3). However, their wide application is restricted by the limited serotypes, high cost, serotype replacement, etc. Compared with pneumococcal conjugate vaccines, pneumococcal whole-cell vaccines may be a better choice for developing countries, since they retained whole molecules which act as pathogen-associated molecular patterns recognized by host antigen-presenting cells and are presented in their natural configuration (9). Various procedures are used for vaccination, even for live-attenuated vaccines. In an investigation of the live-attenuated cholera vaccine VCUSM21P, administration twice at an interval of 14 days yielded acceptable protection (26). Other studies have found that live-attenuated hepatitis A vaccines H2 and LA-1 virus strains exert satisfactory protection with a single dose administered by subcutaneous injection (27). Based on our previous exploration of SPY1 vaccination, we observed a robust humoral and cellular immune response after intranasal vaccination with SPY1 four times, inducing robust immune responses (11, 28). In our previous work, the differences in immune responses and protective efficacies of SPY1 by intraperitoneal immunization and intranasal immunization were evaluated. Intraperitoneal immunization and intranasal immunization both induced elevations in IL-10, IL-4, and IL-17A; however, the levels of IL-17A in splenocyte supernatants and nasal washes as well as secretory IgA in saliva in intranasally immunized mice were significantly higher than those in intraperitoneally immunized mice, suggesting better protection against pneumococcal colonization in an intranasal immunization model (10). In addition, intranasal immunization provided increased protection against lethal pneumococcal challenge at 3 months post-vaccination, and the survival rates for the intranasal immunization model and intraperitoneal immunization model were 100 and 75%, respectively, and this difference can probably be explained by the more rapid clearance of the subcutaneously injected vaccine (29).

Vaccine adjuvants can be used to enhance immunogenicity, accelerate the immune response, increase the duration of protection, and so on (30). For live-attenuated vaccines, some studies have shown that the antigenicity and immunogenicity of vaccine strains, such as live-attenuated *Yersinia pestis* against pneumonic plague are strong enough so that the adjuvant is not necessary (31). However, other studies have shown the indispensable role of adjuvants for vaccination with live-attenuated vaccines, such as the cholera vaccine candidate VCUSM21P (26) and H5N1 live-attenuated influenza vaccines (32). In our previous research, we have evaluated the vaccination of SPY1 without adjuvant CT; however, the protective effect was not as efficient as that obtained by CT (data not published). We used adjuvant CT, as in our other studies of SPY1 (10, 11). However, the toxicity of CT limits its application in humans, making the development of safe and nontoxic adjuvants that induce protective immunity essential for the nasal immunization of SPY1 in humans. We have developed safe and effective mucosal adjuvants as CT alternatives, such as the mast cell activator compound 48/80 (C48/80) (33) and CaPi mineralized shell (34). Coupled with these nontoxic adjuvants, protection induced by SPY1 or its derived vaccine SPY1ΔlytA is as efficient as that obtained with CT, ensuring the future application of SPY1 in humans.

The balance between immune response activation essential for host defense and immune suppression restricting excessive host damage caused by the immune response should be strictly regulated during pathogen invasion. In this study, P17, which downregulates Tregs, influenced the systemic protective immune response elicited by SPY1 immunization. P17 treatment significantly upregulated levels of the infection-associated inflammatory cytokine TNF-α and immune cytokines (IL-12p70, IFN-γ, IL-4, IL-5, and IL-17A) and downregulated concentrations of the immunosuppressive cytokine IL-10 and inflammatory mediator IL-6 (23) secreted by splenocytes of SPY1-immunized mice. Correspondently, compared with SPY1-immunized mice not treated with P17, we observed more severe pulmonary injuries in P17-treated immunized mice during pneumococcal infection. Moreover, the changes in cytokines in immunized mouse lung homogenates were in line with those in splenocytes mentioned above. These data indicated that the SPY1-specific Treg immune response could inhibit uncontrolled pulmonary immune responses and infection-associated inflammation and thereby limit excessive immunopathology in immunized mice, resulting in acquired immune homeostasis, which is necessary for vaccination-induced protection against pneumococcal infection. P17 suppressed the increase in IFN-γ; however, the elevation of IFN-γ in immunized mice was not SPY1-specific (no significant differences in the IFN-γ increase were detected between the CT group and CT + SPY1 group) and were probably caused by CT, consistent with the results of our previous study (11) and other reports (35). As a crucial pro-inflammatory cytokine (36), IL-12p70 is elevated by SPY1-infected dendritic cells co-cultured with CD4^+^ cells (37), consistent with the results of this study. Several studies have suggested that IL-6 is a pro-inflammatory cytokine in infection (38); however, other studies have shown that IL-6-deficient mice exhibit impaired resistance against *S. pneumoniae* (23), *Listeria monocytogenes* (23, 39), and *Escherichia coli* (40), implying that IL-6 inhibits inflammation. Taken together, these findings suggest that IL-6 is a dual cytokine. As one of the immunosuppressive cytokines secreted by Tregs, the roles of IL-10 in infection are unclear. IL-10 impairs the immunosuppressive activity of Tregs in murine models of schistosomiasis japonica or asthma (41). However, during remission stage in mice with lymphocytic choriomeningitis virus infection, IL-10 produced by Tregs promotes the maturation of memory CD8^+^ T cells, which is beneficial for host defense against secondary infections by intracellular pathogens (42). Similarly, in this study, dramatically elevated expression of IL-10 and palliative pulmonary injuries was observed in SPY1-immunized mice, indicating the beneficial effect of IL-10 on immunoprotection of SPY1-specific Tregs.

There are three main types of CD4^+^ regulatory cells, i.e., Tr1, CD4^+^ Th3, and CD4^+^CD25^+^Foxp3^+^ T cells (43). Most studies have focused on CD4^+^CD25^+^Foxp3^+^ T cells, which are indispensable for the maintenance of the immunologic balance (44). SPY1 could induce the Treg immune response involved in protection against pneumococcal infection (11). In this study, we showed that the expression of Foxp3, a key transcription factor belonging to the Treg immune pathway, mediates the activation of SPY1-specific Tregs. Obviously upregulated Foxp3 attributed to SPY1 vaccination was detected, suggesting that CD4^+^CD25^+^Foxp3^+^ T cells are the major type of SPY1-specific Tregs.

Regulatory T cell differentiation is mainly regulated by TGF-β/Smad signaling (24). Smad2 and Smad3 contribute to Foxp3 induction *via* different mechanisms, Smad3 directly interacts with an enhancer region of *Foxp3*, i.e., CNS1 (45), and the interaction is believed to be necessary for sustaining normal Foxp3^+^ Treg numbers in the mouse gut but not in other organs (46). Smad2 has a relatively lower affinity to DNA than Smad3, and it cannot directly interact with CNS1 (47). However, the absence of Smad2 significantly decreases the upregulation of Foxp3 on T cells (48) and the deletion of both Smad2 and Smad3 could completely abolish the induction of Foxp3 by TGF-β (49), illustrating the synergetic effects of Smad2 and Smad3 in Treg induction. We observed increased levels of Smad2 and Smad3 as well as decreased levels of the negative regulatory factor Smad7 in the SPY1 vaccination group. These changes in Smads were significantly reversed by P17 treatment due to the inhibition of TGF-β1, illustrating that SPY1-stimulated TGF-β1 induced the generation of SPY1-specific Tregs *via* the Smad2/3 signaling pathway. Given that several Smad-independent pathways are also responsible for the induction of Tregs *via* TGF-β in different experiment models (18–21), the levels of *p38 mapk, akt, mtor*, and *pi3k* in pulmonary tissues were determined by real-time PCR, and no significant differences were detected among the control group, P17-treated SPY1-immunized group, and non-P17-treated immunized group (data not shown). Nevertheless, we cannot rule out the potential roles of other unknown signaling pathways in the generation of SPY1-specific Tregs for the immunoprotection in mice.

As foremost costimulatory inhibitors, PD-1 and CTLA-4 play key roles in the suppressive activity of Tregs (25, 50). We observed enhanced expression of PD-1 and CLTA-4 on SPY1-specific Tregs and their subsequent roles in immunoprotection elicited by SPY1-specific Tregs together with increased IL-10. TGF-β is believed to be another important inhibitory cytokine secreted by Tregs; however, its suppressive function remains contentious (47). Some research has revealed that TGF-β1 is redundant for Treg suppressor functions (51), but Ming et al. (52) showed that Treg-derived TGF-β1 is essential for controlling inflammatory-bowel disease. Therefore, the role of TGF-β1 in the immunoprotective function of SPY1-specific Tregs may be complicated.

Despite their crucial role in immune homeostasis and in the prevention of autoimmunity, the function of Tregs in infection is still controversial. Tregs are detrimental to hosts by restricting the effector T cell immune response during early tuberculosis (53). In some cases, virus or bacteria-specific Tregs not only prevent pathogen elimination but also promote a generalized state of immune suppression *in vivo*, making the host more susceptible to secondary infections with other pathogens (54). However, growing evidence has suggested that interactions between pathogens and Tregs are mutually beneficial to the pathogen and host, which allows persistent infection, conferring the maintenance of long-term memory and resistance to reinfection in a model of *Leishmania major* infection (55). Furthermore, emerging evidence suggests that Tregs can be beneficial to the host by restricting an overly robust inflammatory response, which causes excessive collateral damage to self-tissues (56), and by promoting the reparation of damaged tissues (57). Accordingly, Tregs should be established to regulate immune responses by maintaining homeostasis for efficient vaccination (58), highlighting the importance of considering vaccine-induced protective Tregs in the design of vaccine candidates.

In addition to Tregs and the regulatory cytokines IL-10 and IL-6, other cells including myeloid-derived suppressor cells, regulatory B cells (Bregs), regulatory γδ T cells, and immunosuppressive plasmocytes, as well as the cytokine IL-35 have immunosuppressive functions. Bregs have strong immunosuppressive effects and can negatively regulate immune responses in malignant tumors (59), infections (60), and autoimmune diseases (61) by multiple mechanisms. IL-35 is a vital anti-inflammatory cytokine that not only induces Bregs but also serves as an important effector of Bregs, participating in the regulation of the immune response *via* a positive feedback network. The immunosuppressive effects of Bregs and IL-35 have been found in innate immunity model against infection but not in an acquired immunity model with vaccination, and we cannot exclude the potential roles of other immunosuppressive cells and cytokines in the immune protective effect of SPY1.

As identified using a phage-displayed peptide library, peptide P17 is an effective inhibitor of Tregs by inhibiting TGF-β1, and its effect and specificity on Tregs have been well documented in various models, including in an invasive pneumococcal infection model (17, 62). P17 inhibits TGF-β1, TGF-β2, and TGF-β3 activity according to a previous study (17). Being predominantly expressed in the immune system, TGF-β1 is believed to be a crucial pleiotropic cytokine with potent immunoregulatory properties (24). The binding of P17 to TGF-β1 is stronger than binding to other isoforms of TGF-β (17). Therefore, in this study, we focused on TGF-β1. In follow-up work, the protective effect of SPY1-specific Tregs may be further established by evaluating a co-culture of TGF-β1 with mice splenocytes *in vitro* or by supplying mice with recombinant TGF-β1 *in vivo*. In addition, depletion of regulatory T cell mice (63) can be used to further evaluate the protection and related mechanisms of SPY1-induced acquired Treg immune responses in future studies.

To conclude, we characterized the mechanism underlying the protective role of vaccine-specific Treg immune response in response to the novel live-attenuated pneumococcal vaccine SPY1 in a mouse model. Immunization with SPY1 stimulated the activation of TGF-β1 *via* the Smad2/3 signaling pathway, which led to the production of Foxp3^+^ Tregs and the subsequent upregulation of the costimulatory inhibitors PD-1 and CTLA-4. The activated SPY1-specific Treg immune response maintained the beneficial immune balance among the infection-associated inflammatory cytokine TNF-α, immune cytokines IL-6, IL-12p70, IL-4, IL-5, and IL-17A, as well as the immunoregulatory cytokine IL-10, and further alleviated excessive pulmonary injury, resulting in decreased bacterial colonization, elevated survival rates, and prolonged survival. Our results indicated that a protective immune response is elicited by vaccine-specific Tregs *via* the TGF-β1-Smad2/3 pathway. These findings may contribute to the comprehensive assessment of live vaccines and other mucosal vaccine candidates.

## Ethics Statement

The research was proved by The Ethics Committee of Chongqing Medical University. All the animal experiments were done in accordance with the guidelines of the Institutional Animal Care and Use Committee of Chongqing Medical University.

## Author Contributions

HL, XP, and LG were in charge of the whole project and participated in manuscript drafting. YG, SY, XH, and LG contributed to lab work and data analyses. JF, LZ, HW, YY, and XX revised the paper critically. All the authors have reviewed the manuscript.

## Conflict of Interest Statement

The authors declare that the research was conducted in the absence of any commercial or financial relationships that could be construed as a potential conflict of interest.
