# Supplementary material for: Protective Regulatory T Cell Immune Response Induced by Intranasal Immunization With the Live-Attenuated Pneumococcal Vaccine SPY1 via the Transforming Growth Factor-β1-Smad2/3 Pathway
Source: Front Immunol. 2018 Aug 2;9:1754. doi: 10.3389/fimmu.2018.01754 (PMC6082925; doi:10.3389/fimmu.2018.01754)
Supplement: Supplementary file 1 [file Data_Sheet_1.docx]

**Supplemental Figure 1**





**Supplemental Figure 1.** On day 28, mice were intranasal challenged with 1×10^8^ CFU of pneumococcal strain D39, and survival days were monitored for consecutive 21 days after infection. Each dot represents one mice, mean survival day indicated by horizontal lines. *, *p* < 0.05.

**Supplemental Figure 2**

**
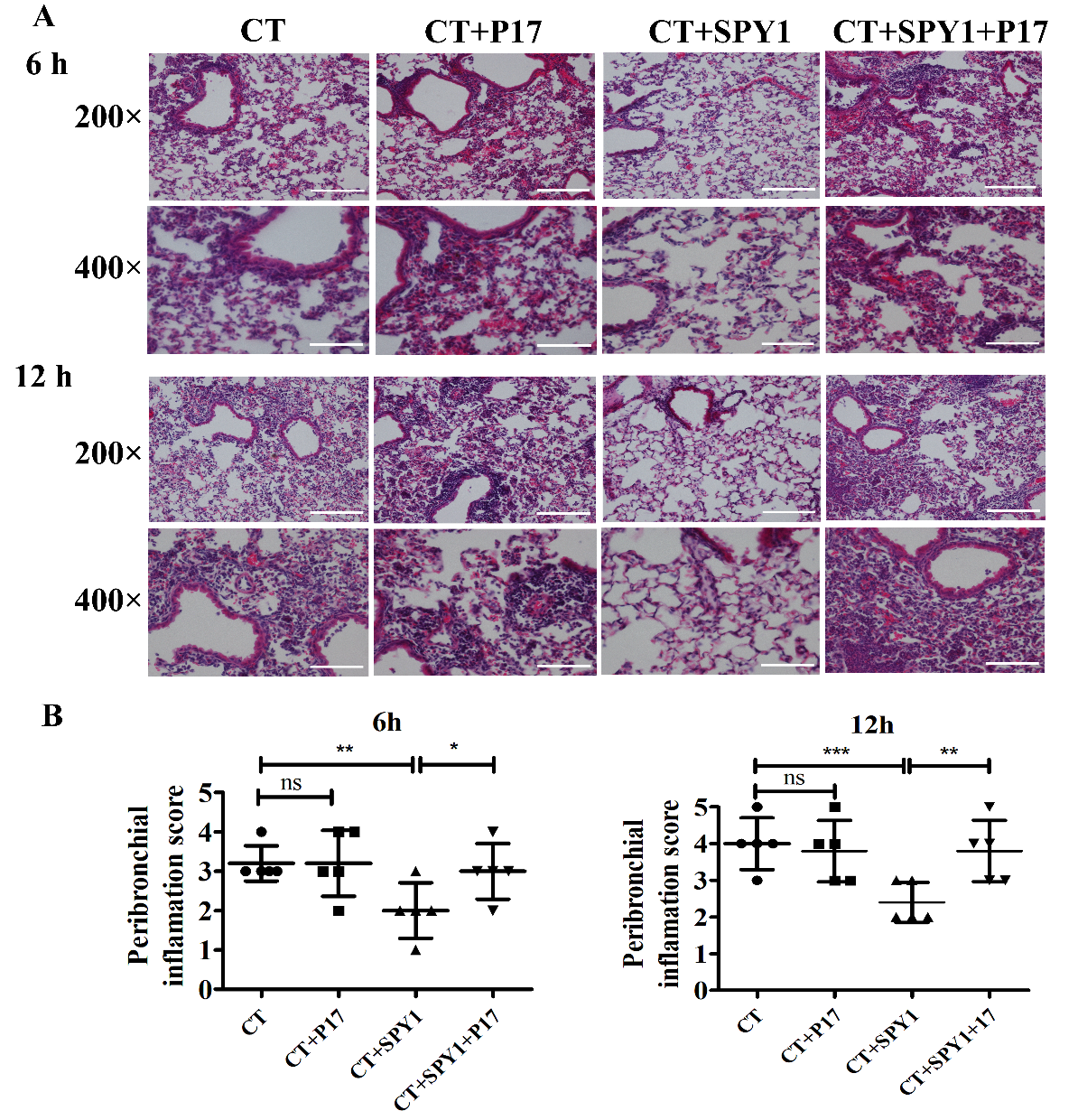
**

**Supplemental Figure 2.** Mice were intranasal challenged with pneumococcal strain 19F on day 7 post the last immunization. **(A)** At 6 h and 12 h post infection, lung tissues were removed and pathological analyses were done by H&E staining, with lung sections examined under light microscopy at magnification 200× (scale bar = 100 μm) and 400× (scale bar = 50 μm), respectively. **(B)** Scores of peribronchial inﬂammations were semi-quantitatively graded, data were shown as mean ± SD of scores of five mice per group. *, *p* < 0.05; **, *p* < 0.01; ***, *p* < 0.001.

**Supplemental Figure 3**

**
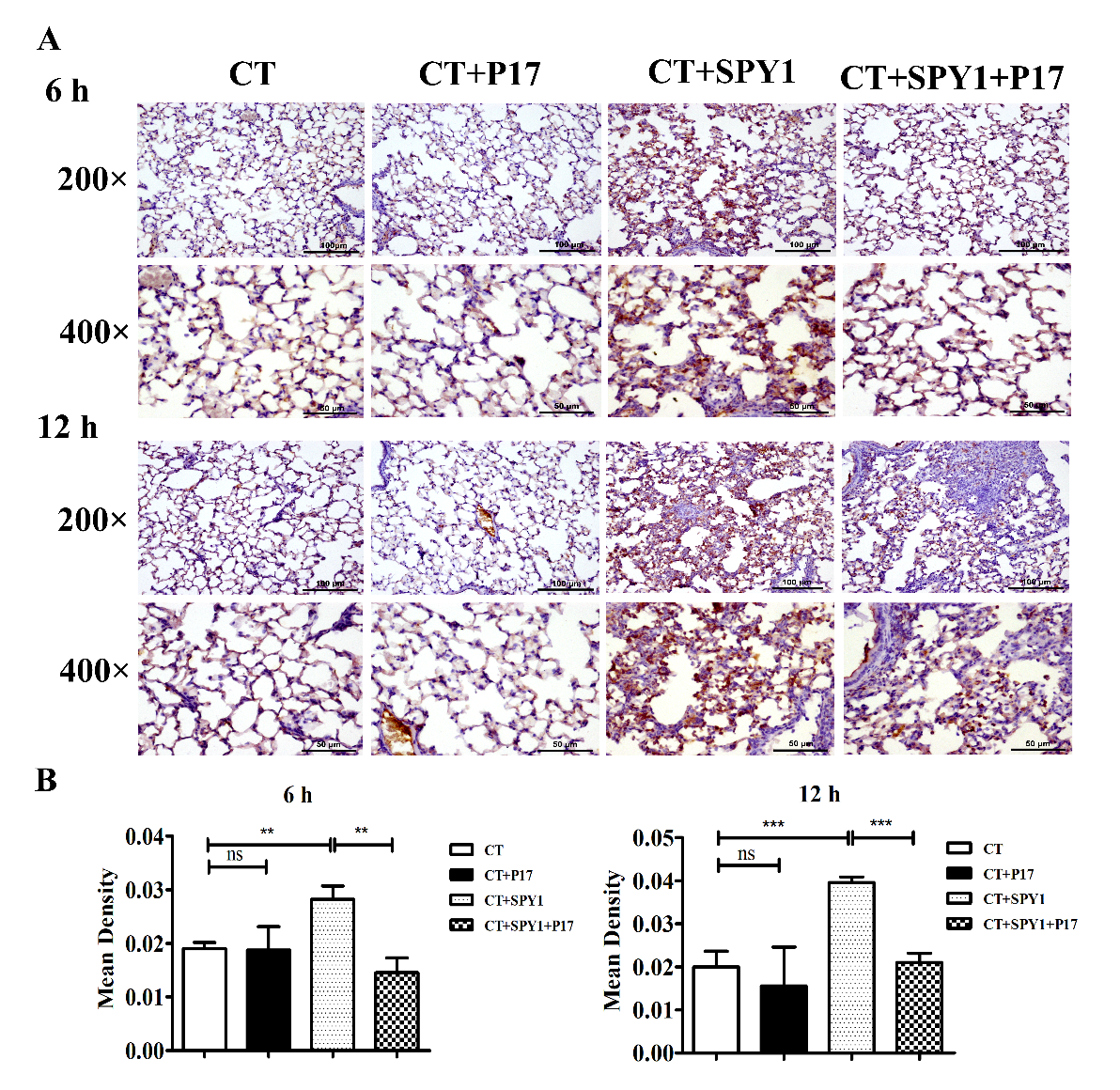
**

**Supplemental Figure 3.** Mice were intranasal challenged with pneumococcal serotype 19F strain on day 7 post the last immunization. **(A)** Productions of TGF-β1 in lungs at 6 h and 12 h post infection were analyzed by immunohistochemical staining, respectively. Sections were examined under light microscopy at magnification 200× (scale bar = 100 μm) and 400× (scale bar = 50 μm). **(B)** The mean IODs of TGF-β1 expression were measured and calculated by Image-Pro Plus. Data were shown as mean ± SD of IODs of five mice per group. **, *p* < 0.01; ***, *p* < 0.001.

**Supplemental Table 1:** Sequences of PCR primers used for real time PCR

|  |  |  |  |
| --- | --- | --- | --- |
| Gene | Orientation | Primer sequence (5′ to 3′) | Product size (bp) |
| *β-actin* | Sense | GCGAGCACAGCTTCTT | 205 |
|  | Anti-sense | TGACCCATTCCCACCAT |  |
|  |  |  |  |
| *foxp3* | Sense | ATGTTCGCCTACTTCAGAA | 173 |
|  | Anti-sense | GGATTGGAGCACTTGTTG |  |
|  |  |  |  |
| *tgf-β1* | Sense | CACTCCCGTGGCTTCTAGTG | 145 |
|  | Anti-sense | GGACTGGCGAGCCTTAGTTT |  |
|  |  |  |  |
| *smad2* | Sense | GGGAGCAGAATATCGGAGGC | 130 |
|  | Anti-sense | GCTTGAGCATCGCACTGAAG |  |
|  |  |  |  |
| *smad3* | Sense | GTCAACAAGTGGTGGCGTGTG | 150 |
|  | Anti-sense | GCAGCAAAGGCTTCTGGGATAA |  |
|  |  |  |  |
| *smad4* | Sense | GGATGGACGACTTCAGGTGG | 291 |
|  | Anti-sense | CCTTCAGTGGGTAAGGACGG |  |
|  |  |  |  |
| *smad7* | Sense | GGAAGATCAACCCCGAGCTG | 125 |
|  | Anti-sense | ACAGCCTGCAGTTGGTTTGAG |  |
|  |  |  |  |
| *p38 mapk* | Sense | GAACAAGACCGTCTGGGAGGTGC | 159 |
|  | Anti-sense | TTGGCGTGAATGATGGACTGAAA |  |
|  |  |  |  |
| *akt* | Sense | ACGCTACTTCCTCCTCAA | 278 |
|  | Anti-sense | TCTCTTCTTCCTGCCTCTT |  |
|  |  |  |  |
| *mtor* | Sense | AGCAACAGTGAGAGTGAAG | 143 |
|  | Anti-sense | AAGGAGATAGAACGGAAGAAG |  |
|  |  |  |  |
| *pi3k* | Sense | CAGGATCAAGTTGTCAAAGAAGAT | 231 |
|  | Anti-sense | TATGTATTCTTTGCTGTACCCCTC |  |
|  |  |  |  |
